# Interim Influenza Vaccine Effectiveness Against Laboratory-Confirmed Influenza — California, October 2023–January 2024

**DOI:** 10.15585/mmwr.mm7308a4

**Published:** 2024-02-29

**Authors:** Sophie Zhu, Joshua Quint, Tomás M. León, Monica Sun, Nancy J. Li, Mark W. Tenforde, Seema Jain, Robert Schechter, Cora Hoover, Erin L. Murray

**Affiliations:** ^1^Epidemic Intelligence Service, CDC; ^2^California Department of Public Health; ^3^Influenza Division, National Center for Immunization and Respiratory Diseases, CDC.

**Summary**

**What is already known about this topic?**

Influenza vaccine effectiveness (VE) is determined using multiple platforms and varies from year to year.

**What is added by this report?**

Using timely surveillance data from mandatory influenza laboratory surveillance and the immunization registry in California, investigators estimated that VE for laboratory-confirmed influenza during October 2023–January 2024 was 45%. VE was highest among persons aged <18 years (56%) and declined with age to 30% among adults aged ≥65 years.

**What are the implications for public health practice?**

Mandatory reporting requirements of laboratory surveillance and vaccination data allow for early season VE estimates while seasonal influenza viruses are circulating. Influenza vaccination remains the best way to prevent influenza, and vaccination is recommended for all persons aged ≥6 months.

## Abstract

Surveillance data can provide rapid, within-season influenza vaccine effectiveness (VE) estimates to guide public health recommendations. Mandatory reporting of influenza vaccine administration to California’s immunization information registry began January 1, 2023, and mandatory reporting of all influenza laboratory test results, including negative results, was instituted in California on June 15, 2023. These data, collected by the California Department of Public Health during October 1, 2023–January 31, 2024, were used to calculate interim influenza VE against laboratory-confirmed influenza by comparing the odds of vaccination among case-patients (persons who received a positive influenza laboratory test result) and control patients (those who received a negative influenza laboratory test result). VE was calculated as 1 − adjusted odds ratio using mixed-effects logistic regression, with age, race, and ethnicity as fixed effects and specimen collection week and county as random effects. Overall, during October 1, 2023–January 31, 2024, estimated VE was 45% among persons aged ≥6 months, 56% among children and adolescents aged 6 months–17 years, 48% among adults aged 18–49 years, 36% among those aged 50–64 years, and 30% among those aged ≥65 years. Consistent with some previous influenza seasons, influenza vaccination provided moderate protection against laboratory-confirmed influenza among infants, children, adolescents, and adults. All persons aged ≥6 months without a contraindication to vaccination should receive annual influenza vaccination to reduce influenza illness, severe influenza, and strain on health care resources. Influenza vaccination remains the best way to prevent influenza.

## Introduction

Each year in the United States, influenza virus is estimated to cause approximately 9–41 million infections, 140,000–710,000 hospitalizations, and 12,000–52,000 deaths (*1*). Vaccination protects against illness, hospitalization, and death associated with influenza (*2*), and annual influenza vaccination is recommended for all persons aged ≥6 months (*3*). However, effectiveness of seasonal influenza vaccines varies by influenza season, the recipient’s age, and other factors (*4*). Mandatory reporting of administration of all influenza vaccine doses to California’s immunization information system (IIS) began January 1, 2023. Positive influenza laboratory test results first became reportable in California on October 1, 2019, and negative results became reportable on June 15, 2023.* The California Department of Public Health (CDPH) used these data sources to estimate early influenza season vaccine effectiveness (VE) against laboratory-confirmed influenza during October 2023–January 2024, including by age and influenza type.

## Methods

VE against laboratory-confirmed influenza was estimated using a case-control design by comparing the odds of current season influenza vaccination among persons who received a positive influenza test result (test-positive case-patients) and persons who received a negative influenza test result (test-negative control patients). All persons who received testing for influenza using molecular nucleic acid amplification tests at laboratory, hospital, pharmacy, ambulatory, or community-based testing facilities in California during October 1, 2023–January 31, 2024, were eligible for inclusion. Testing and vaccination records were linked using fuzzy matching^†^; a person who received two influenza laboratory results ≥1 day apart was considered to have had repeat testing. The earliest positive test result was used to identify influenza case-patients, and the earliest negative test result (among persons who never received a positive test result) was used to identify control patients. Results from laboratories that reported predominantly positive influenza test results (≥50%) on a weekly basis were excluded because negative results were considered likely to be underreported. These excluded results represented approximately 5% of total laboratory reports. A person was considered vaccinated if immunization records from California’s IIS documented receipt of ≥1 dose of seasonal influenza vaccine ≥14 days before testing during August 1, 2023–January 31, 2024. Persons who were vaccinated <13 days before the test date were excluded. VE against laboratory-confirmed influenza was calculated as 100% × (1 − adjusted odds ratio [aOR]), where the aOR is the odds ratio of vaccination among test-positive case-patients compared with test-negative control patients, adjusted for potential confounders. Using mixed-effects logistic regression, estimates were adjusted for age, race, and ethnicity as fixed effects; specimen collection week and county of residence were random effects. Separate analyses were conducted to estimate VE by influenza type (A or B) and by age group. All analyses were performed using R software (version 4.3.1; R Foundation). This activity was reviewed by CDPH and CDC, deemed not research, and was conducted consistent with applicable federal law and CDC policy.^§^

## Results

During October 1, 2023–January 31, 2024, a total of 678,422 influenza laboratory test results meeting study inclusion criteria were reported to CDPH, including 77,501 (11%) positive and 600,921 (89%) negative test results. The median ages of persons who did and did not receive a positive influenza test result were 31 years (IQR = 10–52 years) and 44 years (IQR = 19–68), respectively (Table 1). Among positive influenza test results, 68,716 (89%) were influenza type A, 7,160 (9%) were type B, and results for 1,625 (2%) specimens were unknown or pending. Overall, 190,313 (28%) persons had documented receipt of the 2023–24 influenza vaccine, including 13,905 (18%) persons who received a positive test result and 176,408 (29%) who received a negative result. Most (74,085 of 83,493; 89%) vaccinated adults aged ≥65 years received a preferentially recommended high-dose, adjuvanted, or recombinant vaccine^¶^ (*3*).

**TABLE 1:** Characteristics of persons with and without laboratory-confirmed influenza — California, October 2023–January 2024

Characteristic	Influenza test result, no. (%)		
	Total	Positive	Negative
**Total (row %)**	**678,422 (100.0)**	77,501 (11.4)	600,921 (88.6)
**Median age, yrs (IQR)**	**42 (17–66)**	31 (10–52)	44 (19–68)
Race*			
American Indian or Alaska Native	**2,919 (0.4)**	326 (0.4)	2,593 (0.5)
Asian	**53,419 (7.9)**	6,252 (8.1)	47,167 (7.8)
Black or African American	**40,069 (5.9)**	4,033 (5.2)	36,036 (6.0)
Native Hawaiian or other Pacific Islander	**2,878 (0.4)**	300 (0.4)	2,578 (0.4)
White	**301,779 (44.5)**	29,908 (38.5)	271,871 (45.2)
Multiple races	**1,381 (0.2)**	130 (0.2)	1,251 (0.2)
Other	**131,284 (19.4)**	16,098 (20.8)	115,186 (19.2)
Unknown	**144,693 (21.3)**	20,454 (26.4)	124,239 (20.7)
Ethnicity*			
Hispanic or Latino	**159,676 (23.6)**	21,309 (27.4)	138,367 (23.1)
Not Hispanic or Latino	**386,200 (56.9)**	38,653 (49.9)	347,547 (57.8)
Unknown	**132,546 (19.5)**	17,539 (22.7)	115,007 (19.1)
**Sex**			
Female	**376,814 (55.5)**	41,321 (53.3)	335,493 (55.8)
Male	**301,187 (44.4)**	36,147 (46.7)	265,040 (44.1)
Other	**85 (0)**	2 (0)	83 (0)
Unknown	**336 (0.1)**	31 (0)	305 (0.1)
**Influenza type**			
A	**—**	68,716 (88.7)	—
B	**—**	7,160 (9.2)	—
Unknown	**—**	1,625 (2.1)	—
**Month vaccinated**			
**Received influenza vaccination**	**190,313 (28.1)**	**13,905 (17.9)**	**176,408 (29.3)**
**By month of influenza test result**			
Oct	**11,073 (13.1)**	93 (6.5)	10,980 (13.2)
Nov	**39,497 (25.3)**	1,357 (12.5)	38,140 (26.3)
Dec	**69,884 (30.1)**	7,104 (17.6)	62,780 (32.7)
Jan	**69,859 (33.9)**	5,351 (21.4)	64,508 (35.6)
**Median no. of days from vaccination to test result (IQR)**	**68 (43–93)**	76 (54–98)	67 (42–92)
**Received high-dose, adjuvanted, or recombinant vaccine, among those aged ≥65 yrs^†^**			
Yes	**74,085 (88.8)**	3,510 (87.4)	70,575 (88.8)
No	**9,408 (11.2)**	503 (12.6)	8,905 (11.2)

* Persons of Hispanic or Latino (Hispanic) origin might be of any race but are categorized as Hispanic; all racial groups are non-Hispanic.

^†^ CDC’s Advisory Committee on Immunization Practices recommends that adults aged ≥65 years receive a high-dose, adjuvanted, or recombinant influenza vaccine. https://dx.doi.org/10.15585/mmwr.rr7202a1

Overall, adjusted VE was 45% against receiving either a positive influenza A or B test result, 42% against receiving a positive influenza A test result, and 76% against receiving a positive influenza B test result (Table 2). VE exhibited an age gradient and was highest among persons aged <18 years (56%), 48% among adults aged 18–49 years, 36% among those aged 50–64 years, and was lowest among adults aged ≥65 years (30%). An age gradient was noted for both influenza A and B, although the VE for persons aged ≥65 years for influenza B (54%) was notably higher than for influenza A (29%).

**TABLE 2:** Effectiveness of influenza vaccine against laboratory-confirmed influenza — California, October 2023–January 2024

Influenza type/Age group, yrs	Positive		Negative		VE	
	Total	No. (%) vaccinated	Total	No. (%) vaccinated	Unadjusted % (95% CI)	Adjusted* % (95% CI)
**Influenza A and B^†^**						
**Total**	**75,876**	**13,629 (18)**	**600,921**	**176,408 (29)**	**47 (46–48)**	**45 (44–46)**
<18	**28,914**	3,744 (13)	**147,047**	32,791 (22)	48 (46–50)	56 (54–57)
18–49	**26,435**	3,334 (13)	**189,129**	36,171 (19)	39 (36–41)	48 (46–50)
50–64	**10,861**	2,575 (24)	**96,148**	28,579 (30)	27 (23–30)	36 (33–39)
≥65	**9,666**	3,976 (41)	**168,597**	78,867 (47)	21 (18–24)	30 (27–33)
**Influenza A**						
**Total**	**68,716**	**13,118 (19)**	**600,921**	**176,408 (29)**	**43 (42–44)**	**42 (41–43)**
<18	**25,393**	3,517 (14)	**147,047**	32,791 (22)	44 (42–46)	52 (51–53)
18–49	**23,257**	3,136 (14)	**189,129**	36,171 (19)	34 (31–36)	44 (42–46)
50–64	**10,546**	2,532 (24)	**96,148**	28,579 (30)	25 (22–29)	35 (32–38)
≥65	**9,520**	3,933 (41)	**168,597**	78,867 (47)	20 (17–24)	29 (26–32)
**Influenza B**						
**Total**	**7,160**	**511 (7)**	**600,921**	**176,408 (29)**	**82 (80–83)**	**76 (73–78)**
<18	**3,521**	227 (6)	**147,047**	32,791 (22)	76 (73–79)	79 (76–82)
18–49	**3,178**	198 (6)	**189,129**	36,171 (19)	72 (68–76)	75 (71–75)
50–64	**315**	43 (14)	**96,148**	28,579 (30)	63 (49–73)	67 (55–76)
≥65	**146**	43 (29)	**168,597**	78,867 (47)	53 (33–67)	54 (34–67)

**Abbreviation:** VE = vaccine effectiveness.

* Adjusted for age (using natural cubic spline interpolation), race, and ethnicity as fixed effects and specimen collection week and county of residence as random effects, using mixed effects logistic regression.

^†^ VE for unknown influenza types was not calculated because of small sample size, and unknown influenza type results were excluded from overall influenza A and B VE estimation.

## Discussion

Analysis of state-level California surveillance data from influenza vaccination and laboratory reporting systems indicates that influenza vaccination provided moderate protection against laboratory-confirmed influenza across all age groups during October 2023–January 2024. Influenza immunization has been demonstrated to avert complications and severe outcomes associated with influenza, including illness, hospitalization, and death (*2*,*5*); therefore, annual influenza vaccination is recommended for all persons aged ≥6 months (*3*). Measured influenza VE in this analysis was highest among children and adolescents and lowest among older adults, a finding that has been observed in previous seasons (*6*). This finding suggests that influenza vaccination for adults aged ≥50 years is less effective than among other age groups. Vaccination of adults aged ≥50 years remains a high priority given their increased risk for severe influenza, even if estimated VE is lower for older adults compared with younger persons (*6*).

Influenza vaccination and laboratory reporting requirements allowed for early season California VE estimates using routine surveillance data. Although the outcome of interest in this analysis (positive influenza laboratory test result) is not one used by CDC VE platforms, the results of this study can be interpreted alongside those from established platforms to broaden characterization of VE. In addition, these VE estimates are consistent with current CDC VE estimates and previous season VE calculations of 40%–60% when influenza viruses are well matched to influenza vaccine components.** Integrated community influenza surveillance and immunization data were used to calculate early season VE estimates during the current (2023–24) influenza season in Canada, and this approach might be feasible for jurisdictions that have similar availability of immunization and laboratory surveillance data (*7*).

In-season early VE estimates that reflect protection against any positive influenza test result can complement established methods that estimate VE against other outcomes or in other settings (*8*). For example, during a year when mismatched influenza viruses predominate, knowledge of low VE might support communication surrounding additional protective and treatment measures against influenza (e.g., social distancing, promoting proper cleaning and disinfection procedures, and use of antivirals) when indicated. Preparations in response to low VE might be especially important in hospitals and long-term care facilities, including measures such as active surveillance for acute respiratory illness among residents and limiting personal contact (*9*). Earlier interim VE estimates might also help guide midseason disease forecasting by providing timely updates of a critical modeling parameter.

### Limitations

The findings in this report are subject to at least seven limitations. First, 2023 was the first year during which reporting of negative influenza test results to CDPH and influenza vaccination to a centralized IIS was mandated. This limitation might have resulted in incomplete reporting and potential bias in observed VE. Second, it was not possible to determine whether infants and children aged <9 years, for whom 2 doses of seasonal influenza vaccine are recommended during their first influenza season (*5*), were fully or partially vaccinated. Third, accompanying symptom information was not available for any influenza laboratory results to differentiate VE against asymptomatic infection or symptomatic illness. Estimates might be biased if characteristics of persons who were tested and those who were not differ. Fourth, information on testing setting (e.g., outpatient, inpatient, or intensive care unit) and illness severity was not available to measure VE for specific outcomes (e.g., hospitalization or death). Fifth, these findings are limited to California and might not be generalizable across the entire United States; multiple influenza viruses circulate, and the proportion of circulating viruses and testing practices by area might differ, as might VE against each influenza virus type or subtype. Sixth, subtype information was not available to estimate VE against influenza A(H1N1)pdm09 and A(H3N2) viruses, though >80% of influenza A strains characterized this season at California public health laboratories are H1N1(pdm09), similar to national data.^††^ Finally, other sources of confounding and bias were not assessed, including the presence of preexisting conditions in the study population that might affect the likelihood of severe influenza, propensity to seek health care and influenza testing, or influenza vaccination behaviors that are differential with respect to infection status (*10*).

### Implications for Public Health Practice

Surveillance data can be used to calculate interim VE quickly and efficiently at the state level. Early VE estimates might allow for more timely public health action for influenza prevention and treatment recommendations. Interim VE estimates from this analysis indicate that the current seasonal influenza vaccine protects against receipt of a positive influenza laboratory test result among persons aged ≥6 months. Annual influenza vaccination is recommended for all persons aged ≥6 months to prevent illness and adverse complications associated with influenza. Influenza vaccination remains the best way to prevent influenza.

## Footnotes

*
https://www.cdph.ca.gov/Programs/CID/DCDC/CDPH%20Document%20Library/Laboratory_Reporting_Letter_COVID_Influenza_RSC_Sept2023_Final.pdf

† California influenza testing and immunization registries were matched using a probabilistic algorithm with exact match for date of birth and fuzzy match with a 95% cutoff on first name, last name, and county of residence.

§ 45 C.F.R. part 46, 21 C.F.R. part 56; 42 U.S.C. Sect. 241(d); 5 U.S.C. Sect. 552a; 44 U.S.C. Sect. 3501 et seq.

¶ CDC’s Advisory Committee on Immunization Practices recommends that adults aged ≥65 years receive a high-dose, adjuvanted, or recombinant influenza vaccine.

**
https://www.cdc.gov/flu/vaccines-work/vaccineeffect.htm

††
https://www.cdph.ca.gov/Programs/CID/DCDC/CDPH%20Document%20Library/Immunization/Week2023-2407_FINALReport.pdf
